# *Hspb1* and *Tp53* Mutation and Expression Analysis in Cat Mammary Tumors

**DOI:** 10.15171/ijb.1480

**Published:** 2016-09

**Authors:** Rashid Saif, Ali Raza Awan, Leslie Lyons, Barbara Gandolfi, Muhammad Tayyab, Masroor Ellahi Babar, Asim Khalid Mehmood, Zia Ullah, Muhammad Wasim

**Affiliations:** ^1^Department of Biotechnology, Virtual University of Pakistan, Lahore 54000, Pakistan; ^2^Institute of Biochemistry and Biotechnology, University of Veterinary and Animal Sciences, Outfall Road, 5400, Lahore, Pakistan; ^3^Department of Veterinary Medicine and Surgery, College of Veterinary Medicine, University of Missouri-Columbia, Columbia, MO 65211, USA; ^4^Pet Center, University of Veterinary and Animal Sciences, Outfall Road, 5400, Lahore, Pakistan

**Keywords:** Cat mammary tumor, *Hspb27* mutation and expression, *Tp53* mutation and expression

## Abstract

**Background:**

Molecular marker based cancer diagnosis gaining more attention in the current genomics era. So, *Hspb1* and *Tp53* gene characterization and their mRNA expression might be helpful in diagnosis and prognosis of cat mammary adenocarcinoma. It will also add information in comparative cancer genetics and genomics.

**Objectives:**

Eight tumors of Siamese cats were analyzed to ascertain germ-line and tissue-specific somatic DNA variations of *Hspb1* and *Tp53* genes along with the ectopic differential expression in tumorous and normal tissues were also analyzed.

**Materials and Methods:**

Tumorous tissues and peripheral blood from mammary adenocarcinoma affected Siamese cats were collected from the Pet center-UVAS. DNA and RNA were extracted from these tissues to analyze the *Hspb1* and *Tp53* DNA variants and ectopic expression of their mRNA within cancerous and normal tissues.

**Results:**

Exon 1 and 3 revealed as hotspots in *Hspb1* gene. The 5´UTR region of the exon1 bear six mutation including 3 transitions, 2 transversion and one heterozygous synonymous transversion in two samples at locus c.34C>C/A. Exon 3 has 1 transversion at c.773A>A/T, 3´UTR of this exon harbor two point mutations at 1868A>T and 2193C>T loci. Intron 2 has two alterations at 1490C>C/T and GTCT4del at 1514. Overall up-regulation of *Hspb1* gene was observed. While exons 3, 4 and 7 of *Tp53* harbor a single variationat c.105A>A/G, c.465T>T/C and c.859G>T respectively. The locus c.1050G>G/A in exon 9 is a heterozygous (G/A) in 3 samples and homozygous (G) in 2 other tumours. Introns 3, 5, 7 and 9 harbor 3, 4, 2 and 7 altered loci respectively. Sixty percent of cancers showed up-regulated trend of *Tp53* gene.

**Conclusions:**

Tumor specific mutations and ectopic expression of *Hspb1* and *Tp53* genes might be helpful in the diagnosis of the mammary lesions and endorse their involvement in cat mammary neoplasm.

## 1. Background

The word “cancer” is so fearsome and attention seeking for victims, practitioners and guardians regardless the type of species afflicted. Mammary adenocarcinoma is the third most common cancer in the cats (1). Eighty percent of the total cases are malignant while 10-20% appeared as benign, sooner or later turn into malignant (2). Malignant tumors are equally lethal in animals as they are in humans and several animal cancers e.g. mammary adenocarcinoma in the cat are the best model for studying human cancer due to the resemblance in the cell morphology, histopathology, risk factors and prognosis (3,4). Mammary tumor is a significant health concern in humans and small animals, so especial emphasis was given to ascertain cancer associated sequence number variant (SNVs) and gene expression profiling of *Hspb1* and *Tp53* genes in this neoplasm (5,6).

Molecular diagnostic biomarkers are getting much attention now in the field of oncology, but still there are few studies regarding the authentication and usage of these markers as screening tools (7). Disease associated mutations may serve as tumor markers for a particular type of neoplasm. It is one of the major research emphases to diagnose cancer earlier through molecular diagnostics methodologies using single novel signature mutation responsible for the disease outbreak or combination of SNPs or specific haplotype might be helpful for its diagnosis.

*Hspb1*gene was characterized in this study which is located on chromosome E3 at 973, 860-975, 895 position, encodes only one transcript of 1411 bp with 3 exons and ciphers 205 amino acids, having 88% and 86% sequence identity with the dog and human counterpart, respectively (8). This protein plays its significant role in many processes of tumor development, especially in the cell cycle regulation, immunosurveillance, cell differentiation, and in the apoptotic pathways. High level of this protein was reported in regression stage of cancer and linked with anti-apoptotic activities (9).

*Tp53* was selected due to being the most variant gene in any type of cancer (10). It is mutated in more than 50% of all malignancies (11). In cats, it is located on E1 chromosome, has only one transcript of 1161 bp with 10 exons, and encrypted with 386 amino acids (12). Tp53 protein behaves as a transcription factor, maintains cell growth and genomic integrity (13,14).

## 2. Objectives

The objectives of the current study are to ascertain cancer associated DNA mutations and expression profiles in *Hspb1*and *Tp53* genes in cancer and disease free controls. A sensitive and robust, endpoint conventional long-range PCR technique was used to characterize these genes using “Sequencher” software and gene expression profiling through RT-qPCR by TaqMan assay chemistry, which will give us better insight to understand genetic variations and gene expression data simultaneously in cat mammary cancer to improve its clinical diagnosis.

## 3. Materials and Methods

### 3.1. Sample Collection

Six mammary tumor tissues and peripheral blood of affected Siamese cat including one normal domestic random bred cat were collected through standard protocol (Table 1). All neoplastic tissues were excisional biopsies. All tissue masses were storedin -86ºC for DNA/RNA extraction and downstream processes (15).

### 3.2. DNA and Total RNA Extraction

TaiGen genomic DNA tissue kit (TaiGen Biotechnology Co., Ltd, Neihu Dist., Taipei, Taiwan) was used to extract DNA from the tumorous tissues (16). While genomic DNA from blood was extracted using GF-1 tissue blood combi DNA extraction kit (Vivatis Technologies SDN. BHD. Selangor Darul Ehsan, Malaysia). DNA quantification was done using NanoDrop spectrophotometer (Thermo Scientific, Wilmington, DE, USA). 50 ng.μL^-1^ concentration of DNA was used for downstream PCR amplifications.

Similarly, total RNA was extracted from cancerous and normal tissues using Thermo Scientific GeneJet RNA purification kit (17) following to the pulverization of the tissues in the liquid nitrogen. TriZol reagent method was also used to extract total RNA from minute tissues (18). RNA integrity was confirmed by agarose gel electrophoresis and concentration was measured by NanoDrop spectrophotometer.

### 3.3. Primer and Probes

Long-range primers were designed from DNA sequence ID ENSFCAT00000026034 and ENSFCAT00000009625 for *Hspb1* and *Tp53* through the application of Primer3 and NetPrimer software (PREMIER Biosoft International, Palo Alto, CA) (19,20). Primer express software (Applied Biosystem, USA) was used to design the primer-probe sequences of the *GAPDH* gene as an endogenous control for normalization (21). *Hspb1*and *Tp53* primer-probes expression assays were purchased with FAM flourophore while *GAPDH* probe was labeled with VIC reporter dye on 5´ end and TAMRA as a quencher on the 3´ (Table 2).

### 3.4. PCR Amplification

Long-range PCR was performed using Applied Biosystem thermocycler at 94ºC temperature for 2 min as initial denaturation, then 10 cycles at 94ºC as cyclic denaturation for 10 sec, annealing at 61v for 30 sec and extension temperatures at 68ºC was adopted for 3 and 5 min because the product size were 2303 and 3610 bp in *Hspb1* and *Tp53* genes respectively. Later on, 30 cycles were run with annealing at 59ºC an extension was done with an increment of 20 sec per cycle. The final extension was done with at 72ºC for 5 min. Long-range PCR kit was used, which has high-fidelity polymerase with final concentration of 1.8 U, PCR 10X enhancer-A with final concentration of 1X, PCR additive 5% dimethyl sulfoxide (DMSO) for GC-rich region amplification and 10X reaction buffer with final concentration of 1X (22,23).

### 3.5. Gel Electrophoresis and Data Analysis

**Figure 1:**
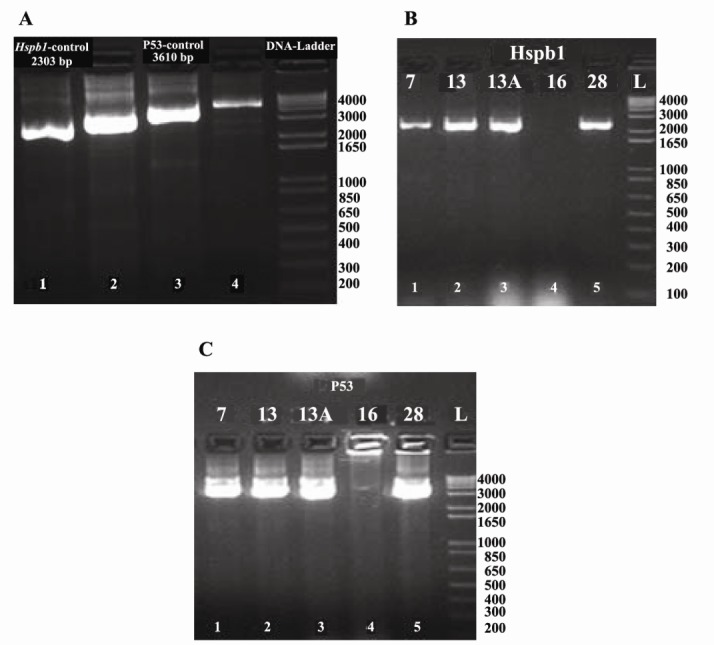
Long-range PCR amplified products of the *Hspb1* and *Tp53* genes in mammary tumor samples and control with 1 Kb Gene Ruler DNA ladder as the size marker. A: Wells number 1 and 3 have product size of 2303 bp and 3610 bp of the *Hspb1* and *Tp53* genes respectively in normal control, B: while a specific product with the size of 2303 bp of *Hspb1* gene in tumor samples are shown in wells 1, 2, 3, 5. C: The PCR amplicon with the 3610 bp product of the *Tp53* gene in tumor samples is shown in wells numbered 1, 2, 3, 5

Electrophoresis with 1.5% agarose gel was conducted for 50 min (Figure 1). Post PCR specific products were purified by treating with ExoSAP (24) (ExoSAP-IT PCR Product Clean up, Santa Clara, CA, USA). Sequencing was done with ABI BigDye termi-nator sequencing Kit (Applied Biosystems, Foster City, CA, USA). “Sequencher” 5.1 software (Gene Codes Corporation, Ann Arbor, MI, USA) was used for sequence analysis (25) (Sequencher® version 5.2 sequence analysis software, Gene Codes Corporation, Ann Arbor, MI USA).

**Table 1:** The signature of the exonic/intronic mutations of the *Hspb1* gene in the mammary tumors of the Siamese cat

**Animal ID**	**Age** **(Yr)**	**Animal /Tissue Type**	**Exon 1**						**Intron 1**	**Intron 2**		**Exon 3**	**3'UTR**	**3' Flanking**
			**-338** **C>T**	**-305** **T>C**	**-288** **G>A**	**-286** **A>G**	**-166** **T>A**	**c.34** **C>A**	**1326** **T>C**	**1490** **C>G**	**1514-1517** **Del** **GTCT**	**c.773** **A>T**	**1868** **A>T**	**2193** **C>T**
Reference	-	Normal	C	T	G	A	T	C	T	C	TTCT	A	A	C
Normal (Domestic random bred)	Un-known	Control/Blood	C	C	A	G	T	C	T/C	C/G	GTCT	A	A	C
CP7	7	Case/Tumor	T	C	G	G	A	C/A	T	G	Del	A	A	C/T
CP13	6	Case/Tumor	C/T	T/C	G	A/G	T/A	C	T/C	C	GTCT	A/T	A/T	C
		Case/Blood	C/T	T/C	G	A/G	A	C	T/C	C	GTCT	A	A	C
CP13A	8	Case/Tumor	C/T	T/C	G	A/G	T/A	C	T/C	C	GTCT	A/T	A/T	C
CP16	7	Case/Tumor	C/T	T/C	G	A/G	T/A	C/A	T	C/G	+/-	A	A	C
		Case/Blood	C/T	T/C	G	A/G	A	C/A	T	C/G	+/-	A	A	C
CP28	2	Case/Tumor	T	C	G	G	A	C	T	G	Del	A	T	C/T
Amino acid change		-						(Cys) Synonymous						

**Table 2:** The signature of the exonic/intronic mutations of the *Tp53* gene in mammary tumor of the Siamese cat

**Animal ID**	**Age** **(Yr)**	**Animal/** **Tissue Type**	**Intron 1**	**Intron2**	**Exon 3**	**Intron 3**			**Exon 4**		**Intron 5**			**Intron 6**			**Exon 7**	**Intron 7**		**Intron 8**			**Exon 9**	**Intron 9**						**3'Flanking**
			**202** **C>G**	**278** **G>C**	**c.105** **A>G**	**769** **C>T**	**776** **C>T**	**958** **Ins (A)**	**c.465** **T>C**	**1474** **T>C**	**1514** **C>T**	**1515** **A>G**	**1555** **G>C**	**1756** **C>T**	**Ins** **1990**	**2002** **C>T**	**c.859 G>T**	**2167** **A>G**	**2217** **T>C**	**2334** **C>T**	**2340** **G>A**	**2415** **T>C**	**c.1050** **G>A**	**2476** **C>T**	**2521** **G>A**	**2737** **G>A**	**2854** **G>A**	**2941** **G>A**	**2943** **A>G**	**3051** **G>A**	**3320** **T>C**
Reference (Abyssinian)	-	Normal	C	G	C	C	C	-	T	T	C	A	G	C	-	C	G	A	T	C	G	T	G	C	G	G	A	G	A	G	C
Control (Domestic random bred)	Un-known	Normal/ Blood	G	C	G	C	C	A	T	T	T	G	C	T	-	C	G	G	C	C	G	T	A	C	A	A	G	G	G	G	T
CP7	7	Tumor	C/G	G	A/G	C	C	A	T/C	T/C	C	A	C	C	-	C	G	A	T	C	G	T	G/A	C	G	G/A	G	G/A	G	G	C
CP13	6	Tumor	C	G	A	C	C	A	T	T	C	A	G	C/T	-	C	G	A/G	T	C	G	T	G/A	C	G/A	G	G/A	G	A/G	G	T/C
		Blood	C	G	A	C	C	A	T	T	C	A	G/C	C/T	-	C	G	A	T/C	C	G	T	G/A	C	G	G	A	G	A/G	G	T/C
CP13A	8	Tumor	C	G	G	C	C	A	T	T	C	A	G	C/T	-	C	G	A/G	T/C	C	G	T	G/A	C	G/A	G	A	G	A/G	G	T/C
CP16	7	Tumor	C	G	A	C	C	A	T	T	C	A	G	C	-	C	G	A	T	C	G	T	G	C/T	G	G	A	G	A	G	C
		Blood	C	G	A	C/T	C	A	T	T	C	A	G	C	-	C	G	A	T	C	G	T	G	C/T	G	G	A	G	A	G	C
CP28	2	Tumor	G	C	G	C	T	A	C	C	C	A	G	C	T	T	T	G	T	T	A	C	G	C	A	G	G	A	G	A	C
Amino acid change									(Tyr) Synonymous								Non-sense						(Arginine) Synonymous								

**Table 3:** Tabulated representation and calculation of *Tp53* gene expression in *Felis catus*

**Sample ID**	**Well**	**Ct-TP53**	**Min**	**Max**	**Range**	**Stan.** **Dev.**	**Mean**	**Well**	**Ct-GAPDH**	**Min**	**Max**	**Range**	**Stan** **Dev.**	**Mean**	**ΔCt.**	**ΔΔCt**	**Fold-change**
CP7	A1	34.65						A4	30.349								
	A2	34.89	34.65	35.27	0.62	0.26	34.94	A5	33.336	30.35	36.38	6.03	2.46	33.35	1.58	0.97	0.51
	A3	35.271						A6	36.377								
CP13	A7	35.937						B2	37.264								
	A8	36.35	35.94	36.74	0.80	0.33	36.34	B3	37.123	36.97	37.26	0.29	0.12	37.12	-0.78	-1.39	2.62
	B1	36.736						B4	36.97								
CP13A	B5	23.877						B8	31.938								
	B6	24.103	23.88	24.29	0.41	0.17	24.09	C1	31.92	31.87	31.94	0.07	0.03	31.91	-7.82	-8.43	344.57
	B7	24.291						C2	31.869								
CP16	C3	27.429						C6	32.647								
	C4	27.355	27.28	27.43	0.15	0.06	27.36	C7	32.51	32.36	32.65	0.29	0.12	32.51	-5.15	-5.76	54.19
	C5	27.281						C8	32.358								
CP28	D1	25.722						D4	33.453								
	D2	25.391	25.32	25.72	0.40	0.17	25.48	D5	33.21	33.21	33.45	0.24	0.11	33.30	-7.82	-8.43	344.65
	D3	25.324						D6	33.231								
Normal control	D7	24.991						E2	25.034								
	D8	24.893	24.89	25.16	0.27	0.11	25.01	E3	23.879	23.88	25.03	1.16	0.48	24.40	0.61	-	-
	E1	25.16						E4	24.298								

**Table 4:** Tabulated representation and calculation of the Hspb1 gene expression levels in Felis catus

Sample #	Well	Ct-HSPB1	Min	Max	Range	Stan Dev.	Mean	Well	Ct-GAPDH	Min	Max	Range	Stan Dev.	Mean	ΔCt.	ΔΔCt	Fold-change
CP7	A1	35.532						A4	30.249
	A2	34.897	34.40	35.53	1.13	0.46	34.94	A5	33.209	30.25	36.00	5.75	2.35	33.15	1.79	1.25	0.42
	A3	34.404						A6	36								
CP13	A7	27.846						B2	37.264
	A8	28.567	27.85	28.57	0.72	0.30	28.18	B3	37.69	36.97	37.69	0.72	0.30	37.31	-9.12	-9.66	811.62
	B1	28.137						B4	36.97								
CP13A	B5	23.877						B8	31.938
	B6	24.1	23.88	24.29	0.41	0.17	24.09	C1	31.909	31.87	31.94	0.07	0.03	31.91	-7.82	-8.36	327.65
	B7	24.291						C2	31.869								
CP16	C3							C6	32.647
	C4	25.55	25.23	26.08	0.85	0.35	25.62	C7	32.39	32.36	32.65	0.29	0.13	32.47	-6.84	-7.38	167.00
	C5	26.081						C8	32.358								
CP28	D1	25.345						D4	31.564
	D2	25.591	25.35	25.88	0.53	0.22	25.60	D5	31.701	31.56	31.87	0.30	0.12	31.71	-6.11	-6.65	100.22
	D3	25.875						D6	31.867								
Normal Control	D7	24.987						E2	24.886
	D8	25.178	24.77	25.18	0.41	0.17	24.98	E3	23.55	23.55	24.89	1.34	0.63	24.44	0.54	-	-
	E1	24.765						E4	24.878								

### 3.6. Reverse Transcription

Target RNA was reverse transcribed using RevertAid first strand cDNA Synthesis Kit (Thermo Fisher Scientific, Pittsburg, PA, USA) (26). Synthesis of first strand cDNA was performed with oligo (dT) 18 primer and random hexamer primers.

### 3.7. RT-qPCR Detection Chemistry and Experimental Design

TaqMan primer-probe hydrolysis chemistry was adopted by using Applied Biosystem 7500 Real-Time System. Twenty μL reaction volume was used, which contains 1 μL 20X TaqMan assay, 10 μL of 2x TaqMan master mix, 4 μL of cDNA with 5 ng.μL^-1^ concentration, plus 5 μL of RNase-free water. Then 40 cycles of reaction were run for amplification (Applied Biosystem, USA). All reactions were designed using singleplex two-step qPCR. Both targets (Hspb1 and Tp53) and GAPDH genes were amplified in triplicate in cases and controls and folds change were obtained from Ct values.

### 3.8. RT-qPCR Data Analysis

Livak method/ΔΔCt method was used in which fold change expression in cancer (Target) vs. normal samples (Calibrator) and constitutively expressed *GAPDH* (Reference) genes were calculated by the following formula (27).

ΔCt (Test) = Ct (Target)-Ct (Reference)

ΔCt (Calibrator) = Ct (Target)-Ct (Reference)

ΔΔCt = ΔCt (Test)-ΔCt (Calibrator)

Fold Change = 2^-ΔΔCt^

## 4. Results

### 4.1. Hspb1 Mutational Spectrum

The reference control sequence of random bred cat was used to align the sequences of our tumour samples (9). *Hspb1* gene of the *Felis catus* has 86% nucleotide identity and 88% protein homology with the human counterpart. No non-synonymous nucleotide alterations were identified in the DNA from five tumorous tissues or in the DNA isolated from blood. Twelve variants were identified in the UTRs, intronic and 5´ flanking regions, but none of them were noticed in cancer cases or in the control. A 4 bp intronic deletion (GTCT) was identified in three cancer samples (CP7, CP16, Cp28), the normal cat has the same sequence of GTCT, but it is absent in the reference sample. Sample (CP16) showed heterozygosity at this position both in somatic tissues and in the blood DNA, which has 4 bp deletions in one allele, while the other allele is same as the wild type. Two 5´ UTR and one 3´ UTR mutation showed a gain of heterozygosity in the tumor as compared to the DNA extracted from blood sample within the same individual.

**Figure 2:**
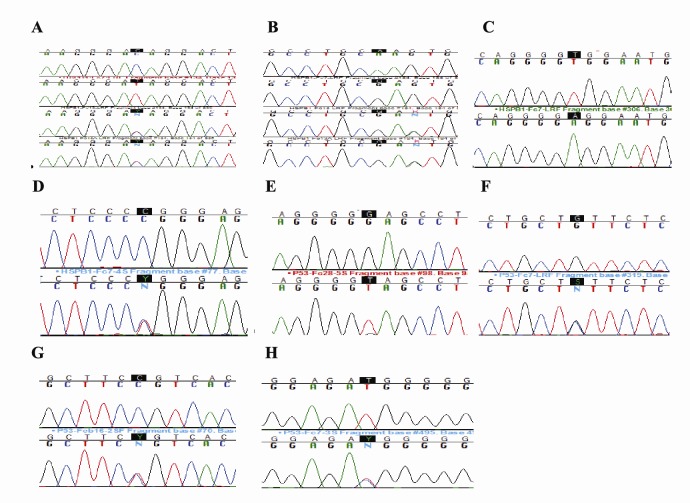
A: Chromatogram of position (-) 338 in exon 1 of *Hspb1* gene in *Felis catus* showing (C/T) heterozygosity in CP13 and CP13A samples instead of homozygous (C) and (T) in control and CP7 respectively. B: Chromatogram of position (-) 288 showing homozygous (G) in CP7, CP13 and CP13A samples instead of homozygous (A) in control, while position (-) 286 in the same exon showing (A/G) heterozygosity in CP13 and CP 13A instead of homozygous (G) in control and CP7. C: Chromatogram of position (-) 166 showing homozygous (A) in CP7 sample instead of homozygous (T) in control. D: Chromatogram of position 2193 in 3´ flanking region showing (C/T) heterozygosity in CP7 sample instead of homozygous (C) in control. E: Chromatogram of position 859 in exon 7 of *Tp53* gene in *Felis catus* showing homozygous (T) in CP28 sample instead of homozygous (G) in control. F: Chromatograms of position 202 in intron 1 of *Tp53* gene in showing heterozygous (C/G) in CP7 sample instead of homozygous (G) in control. G: Chromatogram of position 769 in intron 3 of *Tp53* gene showing heterozygous (C/T) in CP16b sample instead of homozygous (C) in control. H: Chromatogram of position 1474 in intron 5 of *Tp53* gene in *Felis catus* showing heterozygous (T/C) in CP7 sample instead of homozygous (T) in control

Out of the total ten altered positions excluding UTR and 5´ flanking region, half of the mutation were observed as transversion, while the remaining half appeared as transition mutations (Table 3, Figure 2).

### 4.2. Tp53 Mutational Landscape

The overall gain of heterozygosity was observed in the exon 3, 4, and 9 (Table 4). c.105 locus in exon 3 is homozygous (A) in two samples (CP13, CP16) and heterozygous (A/G) in (CP7), but doesn’t change the amino acid. The exon 4 c.465 locus is heterozygous (C/T) in (CP7) while homozygous (C) in another (CP28) sample. Also, it appears to be synonymous. Locus c.859 in exon 7 is homozygous (T) in one sample (CP28) and appeared asa non-sense mutation. Similarly, exon 9 is heterozygous (G/A) at position c.1050 in three cases (CP7, Cp13, CP13A) and proved to be synonymous as well.

Different polymorphic sites were observed in each of the introns 1, 2, and the 3´flanking region at 202, 278 and 3320 loci, respectively. Intron 7 has two hotspots at 2167A>A/G and 2217T>T/C. Introns 3, 6, and 8 have three variant positions in each at positions (769,776,958), (1756,1900,2002), and (2334,2340,2415) respectively. Similarly, introns 5 and 9 have different 4 and 7 point mutation correspondingly. Intronic mutations give us clues regarding how somatic mutations accumulate in a micro-evolutionary process of cancer development. Out of the total 28 polymorphic positions in this gene, 4 positions are transversion, while 24 are transitional changes (Table 4, Figure 2).

### 4.3. Differential Expression of Tp53

Two-step singleplex RT-qPCR was conducted on all mammary tumor cancer cases in triplicate and standardized cDNA of 5 ng.μL^-1^ concentration was used as a template, which was prepared from 100 ng.μL^-1^ stock RNA. Fold change difference expression values were obtained by using ΔΔCt/comparative Ct method. Calculations were performed using ΔCt of *Tp53* gene in all mammary tumor samples. Similarly, ΔCt values of *GAPDH* were also calculated in all cancer samples, while ΔCt calibrator was calculated (0.61) from the mean Ct target *Tp53* subtracted from mean Ct reference/ endogenous *GAPDH* of normal diseased free cat tissues.

ΔΔCt values were obtained by subtracting ΔCt target from the ΔCt calibrator, then this value underwent to the negative exponential power of 2, which represents the efficiency of the assay. Finally, differential expression values as fold change were obtained mentioned in the Table 3.

**Figure 3:**
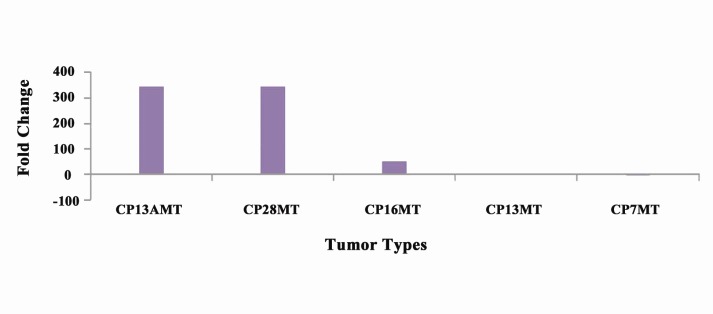
Graphical representation of the *TP53* gene expression in *Felis catus*. The X-axis represents the mammary tumor cases, while the Y-axis shows the differential expression fold change

Four mammary tumors were revealed up-regulated for *Tp53* gene, with the fold change of maximum 344.65, while one sample (CP7) showed down-regulation of this gene (Figure 3).

### 4.4. Hspb1 Differential Expression

Similarly, ΔCt of normal disease-free samples (Calibrator) was calculated (0.54) in Felis catus, which is meant Ct target Hspb1 gene in normal tissues subtracted from the mean Ct of *GAPDH* from the same normal tissues (Table 4).

**Figure 4:**
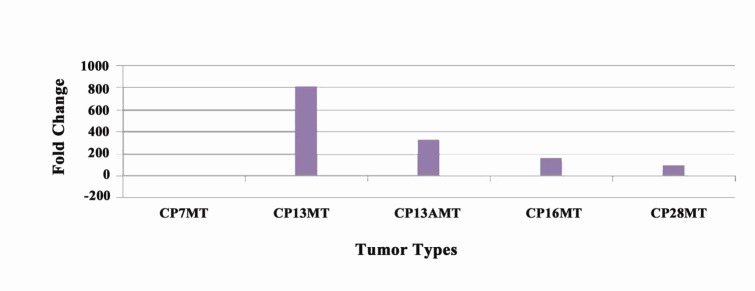
Graphical representation of the *Hspb1* gene expression change in the *Felis catus*. X-axis shows the different mammary tumor cases, while the Y-axis represents the differential expression fold change

Alike *Tp53* up-regulation (in CP13, CP13A, CP16, and CP26), Hspb1 gene is subject of up-regulation in the same samples, while CP7 sample was found to become down-regulated same as *Tp53* (Figure 4).

## 5. Discussion

It is undoubtedly established that accumulation of mutations leads to cancer or cell death. *Hspb1* as a diagnostic marker are not so much informative, but they are expedient indicators for carcinogenesis in some tissues and predict the differentiation and aggressiveness of few cancers (28). This gene has been widely studied in human cancers (29), it has hotspot motif at functional promoter rs2868371 and G1271C (30). Similarly in veterinary species, Austrian feline solid carcinoma of mammary glands, where Arg CGG-TGG Tyr variant was found in exon 8 of *Tp53* (31). The current study did not report this variant probably due to breed or geographical population differences. But it has been found that *Tp53* exon 3, 4, 7 and 9 are more variable as compared to other exons and codon 35, 155, 287 and 350 were found mutant. Codons 35, 155, and 350 are synonymous while the codon 287 terminates the peptide chain and turned into the stop codon.

*Tp53* gene was characterized in FVAS where 8 SNPs along with (T) insertions were found in exon 5, 6, 7 and 8 (32). Codon 163 and positions 14,246, 247, and 259 of intron 7 were also altered (33). The change in the c.105 locus of the *Tp53* in samples CP7 (c.105G>A/G), CP13 (c.105G>A) and CP16 (c.105G>A) were observed (Table 2). The same locus was reported polymorphic in human pancreatic cancer (34), hepatocarcinoma (35), breast and ovarian cancers (36).

Locus c.465C>T of the *Tp53* was found altered in‏ CP7 (c.465T>T/C) and CP28 (c.465T>C) samples‏ (Table 2). Same locus were reported to be altered in‏ eight different studies in human breast cancer (37,38),‏ ovarian cancer (39), hepatocarcinoma (40), colorectal‏ cancer (41), endometrial tumor (42), sinonasal cancer‏ (43), esophageal adenocarcinoma (44), while one‏ study has shown c.465C>A change, which was on‏ esophageal SCC in Chinese population (45). This‏ locus appeared as synonymous in all these studies.‏ Locus c.859 was also found very informative in‏ human studies, as we observed here in cat mammary‏ tumor of CP28 sample, this transversion of‏ (c.859G>T) turned into stop codon in this sample‏ which is a transitional change of (c.859G>A) in DNA‏ binding site of coding strand in five human studies of‏ bladder cancer (46), hepatocellular carcinoma (47),‏ skin SCC (48), aerodigestive tract (49), gastric cancer‏ (50), and changed glutamic acid to lysine (p.E287K) in‏ the protein. Another transversion change of ‏ c.859G->T‏ was also noticed in the five different studies on this‏ locus including Burkitt Lymphoma and chronic lymphocytic‏ leukemia (51), non-small-cell lung cancer‏ (52), bladder carcinoma (53), thyroid carcinoma (54),‏ esophageal carcinomas (55) in which glutamic acid‏ was ‏ changed to stop a codon (p.E287X) in the protein‏ as well as in the present study in this cat mammary‏ tumor (CP28) (Table 2).

The c.1050 position of the *Tp53* in cats mammary‏ tumor was found altered in three cancer samples (CP7,‏ CP13, CP13A), in which c.1050G>G/A change was‏ observed (Table 2), which appeared as synonymous. In‏ one of the human aristolochic acid-associated urothelial‏ cancer in Taiwan population, this locus was also‏ found to be altered at c.1050C->G and synonymous in‏ nature. This point mutation was transversion in the‏ DNA coding strand, which encodes an amino acid in‏ alpha helix structure of this protein (56). Our studied‏ mammary cases have shown alterations in exon 3, 4, 7,‏ and 9 of Tp53 gene. A 4 bp deletion was found in the‏ intron 2 of the Hspb1 gene in the three cat mammary‏ tumors (CP7, CP16, CP28), which is homozygous in‏ two (CP7, CP28), and heterozygous in one neoplastic‏ tissue, as well as the blood of one animal (CP16,‏ CP16b) samples.

### 5.1. Comparison of Hspb1 Polymorphism

Cross-tissue (germ-line vs somatic) mutational comparison of the cat’s Hspb1 gene was re-evaluated and revealed that the mostly altered loci are same in neoplastic tissues and blood of the same animal CP13 and CP16, especially in the exonic regions, but few of the intronic positions show heterozygosity. Similarly, (-)166 locus in 5´UTR of exon 1 in two cases: CP13 and CP16 acquired the same heterozygous (T/A) mutation in tumorous tissues while blood DNA are homozygous (A) at this locus. In sample CP13 at 773 position of 3´UTR of Hspb1 gene found heterozygous (A/T) in cancerous tissues, while homozygous (A) in the blood (Table 1). Other polymorphic loci were observed to be the same in both tissues. Similarly, 3´UTR locus 1868 in CP13 tumorous tissue is also different from the blood. It is homozygous (A) in blood, while heterozygous (A/T) in neoplastic tissues (Table 2).

### 5.2. Comparison of Tp53 Polymorphism

Cross-tissue mutation comparison (germ-line versus‏ somatic) was also conducted for *Tp53*.‏ Comparison of sample ID CP13 and CP16 was conducted‏ which revealed that exonic regions are the same‏ between the two tissue types while five loci in the‏ intronic region in CP13 are different in this crossexamination‏ of polymorphic sites. Few loci in blood‏ are different e.g. gene position 1555 in intron 5 is heterozygous‏ (G/C) in blood instead of homozygous (G)‏ in neoplastic tissue, position 2167 in intron 7 is‏ homozygous (A) in blood instead of heterozygous‏ (A/G) in cancerous tissue. Similarly, two positions‏ 2521 and 2854 in intron 9 are homozygous (G) and (A)‏ instead of heterozygous (G/A) in neoplastic tissues of‏ the sample CP13. CP16 sample was found heterozygous‏ (C/T) instead of homozygous (C) in cancerous‏ tissues in intron 3 at position 769 (Table 2).

Regarding the *Hspb1* and *Tp53* expression in relation‏ to different mutations, up-regulation of *Tp53* was‏ observed in 4 mammary tumors (CP13, CP13A, CP16,‏ and CP28). Two tumors samples (CP13, CP16)‏ showed fold change of 2.62 and 54.19 respectively,‏ while two of the tumor samples (CP13A, CP28)‏ showed almost same up-regulation of 344.57 and‏ 344.65, respectively. Significant up-regulation of *Tp53* expression, as compared to the calibrator, is much‏ more informative in differential diagnosis as compared‏ to those markers which have slightly higher overexpression‏ (Table 3, Figure 3).

*Tp53* up and down regulated samples are significantly‏ different in their mutational landscape, as upregulated‏ samples (CP13, CP16) have 105G>A common‏ mutation in exon 3. Likewise, (CP13, CP13A)‏ have common mutation of 1050G>G/A in exon 9,‏ while up-regulated sample (CP28) was quite different‏ from their counterparts, which has two homozygous‏ variants of 465T>C in exon 4 and 859G>T in exon 7.‏ Different mutations in these four samples which are‏ different from the down-regulated sample are 202C in‏ intron 1, 278G in intron 2, 776C in intron 3, 1474T in‏ intron 5 in (CP13, CP13A, and CP16), while 202G,‏ 278C, 776T, 1474C in (CP28).

Down-regulated sample (CP7) of *Tp53* gene were‏ found heterozygous at 105G>G/A in exon 3,‏ 465T>T/C in exon 4 and 1050G>G/A in exon 9 loci,‏ which are homozygous (A), (T) and (G) in up-regulated‏ samples of the mammary tumors respectively.‏ These loci 202C/G, 769C, 278G, 1474T/C, 1555C,‏ 2737G/A, 2941G/A of (CP7) are different from the‏ above mentioned up-regulated samples (Table 3). Four‏ samples (CP13, CP13A, CP16, and CP28) showed upregulation‏ of the *Hspb1* out of the total five tumors‏ (Table 4, Figure 4). Minimum up-regulation of 100.22‏ fold change was observed in one sample (CP28). One‏ of the tumor sample CP13A showed almost same‏ expression of *Hspb1* and *Tp53* in the range of 327.65‏ and 344.57 respectively. The up-regulation trend of‏ *Hspb1* gene expression can be correlated with common‏ heterozygous mutations of 166T>T/A,‏ 286A>A/G, 305T>T/C, and 388C>C/T in three upregulated‏ samples (CP13, CP13A, and CP16) in exon‏ 1, while (CP28) sample has up-regulation of *Hspb1* gene with all homozygous changes on the same loci‏ 166T>A, 286A>G, 305T>C, and 388C>T.

One of the worth mentioning change was 1514-‏ 1517del4 that has been observed in all four up-regulated‏ samples in intron 2 of *Hspb1*. Similarly,‏ 1326T>T/C change was observed in intron 1 and‏ 1868A>A/T in 3´UTR in two up-regulated samples‏ (CP13, CP13A) while the third up-regulated sample‏ (CP16) was found heterozygous on 1514-1517 locus‏ with additional change of 1490C>C/G in intron 2. The‏ fourth up-regulated sample of (CP28) showed 1514-1517del4 change along with an additional change of‏ 1868A>A/T in 3´UTR. One mammary tumor sample‏ (CP7) showed down-regulation of *Hspb1* as compared‏ to the calibrator, which showed fold change of 0.42.‏ Down-regulation might be associated with homozygous‏ locus of 286A>G, 305T>C and 338C>T. Downregulated‏ sample has homozygous variant on the same‏ three loci as compared to the heterozygous variants in‏ up-regulated samples. These changes are similar to‏ (CP28) but with the difference in one locus of‏ 166T>A. Intronic regions are also found altered in this‏ single down-regulated sample (CP7), in which 1514-1517Del4 and 1490C>G changes were found in the‏ intron 2.

## 6. Conclusions

*Tp53* was found to be more polymorphic than‏ *Hspb1*. Exon 3, 4 and 9 have one synonymous mutant‏ site in each, while one mutant in exon 7 was appeared‏ as non-sense. Introns 1, 2 and 9 were found polymorphic‏ with 1, 1, and 7 variants respectively. Introns 3, 6‏ and 8 have three mutant loci while intron 5 was‏ observed with four mutant loci. Intron 6 has an insertion‏ of 1 bp at the position 1990 in sample CP28.‏ Similarly, exon 1 in *Hspb1* has six polymorphic sites‏ with one synonymous mutation, while remaining five‏ are located in the upstream region. Exon 3 was also‏ observed mutated at a single at genomic position of 1326 in its intronic region. Regarding the gene expression,‏ overall up-regulation of the both genes was‏ observed in this cat neoplasm as compared to normal.

## Acknowledgements

Authors are thankful to HEC-Pakistan, management‏ of University of Missouri-Columbia, USA,‏ Institute of Biochemistry and Biotechnology and Pet‏ center University of Veterinary and Animal Sciences,‏ Lahore-Pakistan.

## References

[1] Lana S, Rutteman G, Withrow S (2007). Tumors of the mammary‏ gland. Small animal clinical oncology.

[2] Giménez F, Hecht S, Craig LE, Legendre AM (2010). Early detection,aggressive therapy: optimizing the management of feline‏ mammary masses. J Feline Med Surg.

[3] Burrai G, Mohammed S, Miller M, Marras V, Pirino S, Addis‏ M (2010). Spontaneous feline mammary intraepithelial lesions‏ as a model for human estrogen receptor-and progesterone‏ receptor-negative breast lesions. BMC Can.

[4] Shafiee R, Javanbakht J, Atyabi N, Bahrami A, Kheradmand D, Safaei R (2013). Comparative value of clinical, cytological, and‏ histopathological features in feline mammary gland tumors; an‏ experimental model for the study of human breast cancer. Diagn Pathology.

[5] Zappulli V, De Zan G, Cardazzo B, Bargelloni L, Castagnaro‏ M (2005). Feline mammary tumours in comparative oncology. J Dairy Res.

[6] Vail DM, Macewen EG (2000). Spontaneously occurring tumors of‏ companion animals as models for human cancer. Cancer Invest.

[7] Hughes K, Dobson J (2012). Prognostic histopathological and molecular‏ markers in feline mammary neoplasia. Vet J.

[8] Fernández XM, Birney E. Ensembl Genome Browser. Vogeland Motulsky’s Human Genetics: Springer; 2010. p. 923-939.

[9] Garrido C, Schmitt E, Candé C, Vahsen N, Parcellier A, Kroemer G (2003). HSP27 and HSP70: potentially oncogenic apoptosis‏ inhibitors. Cell Cycle.

[10] Tomita, Marchenko N, Erster S, Nemajerova A, Dehner A, Klein C (2006). WT p53, but not tumor-derived mutants, bind to Bcl2 via the DNA binding domain and induce mitochondrial‏ permeabilization. J Biol Chem.

[11] Hainaut P, Hollstein M (1999). p53 and Human Cancer: The First Ten‏ Thousand Mutations. Adv Cancer Res.

[12] Kelley MJ, Johnson BE (1994). Genetic mechanisms of solid tumor‏ oncogenesis. Adv in intern med.

[13] Pietsch EC, Sykes SM, McMahon SB, Murphy ME (2008). The p53‏ family and programmed cell death. Oncogene.

[14] Stockmann, Ferrari HF, Andrade AL, Lopes RA, Cardoso TC, Luvizotto MC (2011). Canine transmissible venereal tumors: Aspects‏ related to programmed cell death. Braz J Vet Pathol.

[15] Sambrook J, Russell David W. Molecular cloning: a laboratory‏ manual. Vol. 3: Cold spring harbor laboratory press; 1989.‏.

[16] Vogelstein B, Gillespie, D. USA: Proceedinds National‏ Academy of Science. 1979. Available from: http://www.geneaid.com/sites/default/files/GT5.pdf. 10.1073/pnas.76.2.615 PMC382999 284385

[17] Boom, Sol C, Salimans M, Jansen C, Wertheim-van Dillen P, Van der Noordaa J (1990). Rapid and simple method for purification‏ of nucleic acids. J Clin Microbiol.

[18] Chomczynski. RNA/DNA/Proteins extraction throug TRIzole‏ reagents. 1987. http://tools.lifetechnologies.com/content/sfs/manuals/trizol_reagent.pdf.

[19] Rozen S, Skaletsky H. Primer 3 on the www for general users‏ and for biologist programmers. Bioinformatics methods and protocols: Springer; 1999. p. 365-386. 10.1385/1-59259-192-2:365 10547847

[20] NetPrimer software. Available from: http://www.premierbiosoft.com/netprimer/netprlaunch/Help/xnetprlaunch.html.

[21] Murthi P, Fitzpatrick E, Borg A, Donath S, Brennecke S, Kalionis B (2008). GAPDH, 18S rRNA and YWHAZ are Suitable‏ Endogenous Reference Genes for Relative Gene Expression‏ Studies in Placental Tissues from Human Idiopathic Fetal‏ Growth Restriction. Placenta.

[22] SequelPrep Long PCR kit with dNTPs. Available from:http://www.lifetechnologies.com/us/en/home/references/protocols/nucleic-acid-amplification-and-expression-profiling/pcr-protocol/sequalprep-long-pcr-kit-with-dntps.html.

[23] Innis MA, Gelfand DH, Sninsky JJ, White TJ. PCR protocols:a guide to methods and applications: Access Online via‏ Elsevier; 1990.

[24] ExoSAP-IT PCR Product Cleanup.‏.

[25] Available from: http://www.genecodes.com.

[26] Malek JA, Shatsman S, Akinretoye B, Gill J. Irreversible heat‏ inactivation of DNase I without RNA degradation.

[27] Livak  KJ, Schmittgen  TD (2001). Analysis of Relative Gene‏ Expression Data Using Real-Time Quantitative PCR and the 2- ÄÄCT Method. Methods.

[28] Ciocca DR, Calderwood SK (2005). Heat shock proteins in cancer:diagnostic, prognostic, predictive, and treatment implications. ‏ Cell Stress Chaperones.

[29] Guo, Bai Y, Xu P, Hu Z, Liu L, Wang F (2010). Functional promoter?‏ 1271G> C variant of HSPB1 predicts lung cancer risk‏ and survival. J Clin Oncol.

[30] Lopez JL, Wei Q, Yuan X, Gomez D, Liu Z, Zhuang Y (2011). Functional promoter rs2868371 variant of HSPB1 associates‏ with radiation-induced esophageal toxicity in patients with‏ non-small-cell lung cancer treated with radio (chemo) therapy. Radiother Oncol.

[31] Mayr B, Schaffner G, Kurzbauer R, Schneider A, Reifinger‏ M, Loupal G (1995). Mutations in tumour suppressor gene p53 in two‏ feline fibrosarcomas. Brit Vete J.

[32] Banerji Banerji, Kanjilal S (2006). Somatic alterations of the p53 tumor suppressor‏ gene in vaccine-associated feline sarcoma. Am J Vet Res.

[33] Nambiar PR, Haines DM, Ellis JA, Kidney BA, Jackson ML (2000). Mutational analysis of tumor suppressor gene p53 in feline‏ vaccine site-associated sarcomas. Am J Vet Res.

[34] Casey G, Yamanaka Y, Friess H, Kobrin MS, Lopez ME, Buchler M (1993). p53 mutations are common in pancreatic cancer and are absent in chronic pancreatitis. Cancer lett.

[35] Nishida N, Fukuda Y, Kokuryu H, Toguchida J, Yandell DW, Ikenega M (1993). Role and mutational heterogeneity of the‏ p53 gene in hepatocellular carcinoma. Cancer Res ‏.

[36] Casey G, Lopez ME, Ramos JC, Plummer SJ, Arboleda MJ, Shaughnessy M (1996). DNA sequence analysis of exons 2‏ through 11 and immunohistochemical staining are required to‏ detect all known p53 alterations in human malignancies. ‏ Oncogene.

[37] Glebov OK, McKenzie KE, White CA, Sukumar S (1994). Frequent‏ p53 gene mutations and novel alleles in familial breast cancer. ‏ Cancer Res.

[38] Lai H, Lin L, Nadji M, Lai S, Trapido E, Meng L (2002). Mutations‏ in the p53 tumor suppressor gene and early onset breast cancer. Cancer Biol Ther.

[39] Singer G, Stöhr R, Cope L, Dehari R, Hartmann A, Cao D-F (2005). Patterns of p53 mutations separate ovarian serous borderline tumors and low-and high-grade carcinomas and provide support for a new model of ovarian carcinogenesis: a mutational analysis with immunohistochemical correlation. Am J Surg Pathol.

[40] Iwamoto KS, Fujii S, Kurata A, Suzuki M, Hayashi T, Ohtsuki Y (1999). p53 mutations in tumor and non-tumor tissues‏ of thorotrast recipients: a model for cellular selection‏ during radiation carcinogenesis in the liver. Carcinogenesis.

[41] Boldrini L, Faviana P, Gisfredi S, Donati V, Zucconi Y, Ursino‏ S (2004). Regulation of telomerase and its hTERT messenger in‏ colorectal cancer. Oncol Rep.

[42] Feng Y-Z, Shiozawa T, Horiuchi A, Shih H-C, Miyamoto T, Kashima H (2005). Intratumoral heterogeneous expression of‏ p53 correlates with p53 mutation, Ki-67, and cyclin A expression‏ in endometrioid-type endometrial adenocarcinomas. ‏ Virchows Arch.

[43] Holmila R, Bornholdt J, Suitiala T, Cyr D, Dictor M, Steiniche T (2010). Profile of TP53 gene mutations in sinonasal‏ cancer. Mutat Rese/Funda Mole Mechan Mutage ‏.

[44] Chung SM, Kao J, Hyjek E, Chen YT (2007). p53 in esophageal adenocarcinoma:‏ a critical reassessment of mutation frequency‏ and identification of 72Arg as the dominant allele. Int J Oncol.

[45] Cao W, Chen X, Dai H, Wang H, Shen B, Chu D (2004). Mutational spectra of p53 in geographically localized‏ esophageal squamous cell carcinoma groups in China. Cancer.

[46] Spruck CH, Rideout WM, Olumi AF, Ohneseit PF, Yang AS, Tsai YC (1993). Distinct pattern of p53 mutations in bladder‏ cancer: relationship to tobacco usage. Cancer Res ‏.

[47] Konishi M, Kikuchi-Yanoshita R, Tanaka K, Sato C, Tsuruta‏ K, Maeda Y (1993). Genetic changes and histopathological‏ grades in human hepatocellular carcinomas. Cancer Sci.

[48] McGregor JM, Berkhout RJ, Rozycka M, ter Schegget J (1997). p53‏ mutations implicate sunlight in post-transplant skin cancer‏ irrespective of human papillomavirus status. Oncogene.

[49] Van Rees BP, Cleton-Jansen AM, Cense HA, Polak MM, Clement MJ, Drillenburg P (2000). Molecular evidence of field‏ cancerization in a patient with 7 tumors of the aerodigestive‏ tract. Hum Pathol.

[50] Wang JY, Lin SR, Hsieh JS, Hsu CH, Huang YS, Huang TJ (2001). Mutations of p53 gene in gastric carcinoma in Taiwan. Anticancer Res.

[51] ‏ Gaidano G, Ballerini P, Gong JZ, Inghirami G, Neri A, ‏ Newcomb EW (1991). p53 mutations in human lymphoid malignancies:association with Burkitt lymphoma and chronic lymphocytic‏ leukemia. Proc Natl Acad Sci.

[52] Top B, Mooi WJ, Klaver SG, Boerrigter L, Wisman P, Elbers‏ HR (1995). Comparative analysis of p53 gene mutations and‏ protein accumulation in human non-small-cell lung cancer. Int J Cancer.

[53] Yasunaga Y, Nakanishi H, Naka N, Miki T, Tsujimura T, Itatani H (1997). Alterations of the p53 gene in occupational‏ bladder cancer in workers exposed to aromatic amines Labo‏ investig. A J tech meth and pathol.

[54] Giglia G, Dumaz N, Drougard C, Avril M-F, Daya-Grosjean‏ L, Sarasin A (1998). p53 mutations in skin and internal tumors of‏ xeroderma pigmentosum patients belonging to the complementation‏ group C. Cancer Res.

[55] Lung ML, Chan WC, Zong YS, Tang C, Fok CL, Wong KT (1996). p53 mutational spectrum of esophageal carcinomas from‏ five different geographical locales in China. Canc Epide‏ Biomar & Preven.

[56] Chen C-H, Dickman KG, Moriya M, Zavadil J, Sidorenko VS, Edwards KL (2012). Aristolochic acid-associated urothelial‏ cancer in Taiwan. Proc Natl Acad Sci.

